# Overview of systematic reviews assessing the evidence for shorter versus longer duration antibiotic treatment for bacterial infections in secondary care

**DOI:** 10.1371/journal.pone.0194858

**Published:** 2018-03-28

**Authors:** Igho J. Onakpoya, A. Sarah Walker, Pui S. Tan, Elizabeth A. Spencer, Oghenekome A. Gbinigie, Johanna Cook, Martin J. Llewelyn, Christopher C. Butler

**Affiliations:** 1 University of Oxford, Nuffield Department of Primary Care Health Sciences, Radcliffe Observatory Quarter, Oxford, United Kingdom; 2 University of Oxford, Nuffield Department of Medicine, Experimental Medicine Division, John Radcliffe Hospital, Oxford, United Kingdom; 3 Department of Microbiology and Infection, Brighton and Sussex University Hospitals NHS Trust, Brighton, United Kingdom; 4 Brighton and Sussex Medical School, Department of Global Health and Infection, Falmer, East Sussex, United Kingdom; Natural Environment Research Council, UNITED KINGDOM

## Abstract

Our objective was to assess the clinical effectiveness of shorter versus longer duration antibiotics for treatment of bacterial infections in adults and children in secondary care settings, using the evidence from published systematic reviews. We conducted electronic searches in MEDLINE, Embase, Cochrane, and Cinahl. Our primary outcome was clinical resolution. The quality of included reviews was assessed using the AMSTAR criteria, and the quality of the evidence was rated using the GRADE criteria. We included 6 systematic reviews (n = 3,162). Four reviews were rated high quality, and two of moderate quality. In adults, there was no difference between shorter versus longer duration in clinical resolution rates for peritonitis (RR 1.03, 95% CI 0.98 to 1.09, I^2^ = 0%), ventilator-associated pneumonia (RR 0.93; 95% CI 0.81 to 1.08, I^2^ = 24%), or acute pyelonephritis and septic UTI (clinical failure: RR 1.00, 95% CI 0.46 to 2.18). The quality of the evidence was very low to moderate. In children, there was no difference in clinical resolution rates for pneumonia (RR 0.98, 95% CI 0.91 to 1.04, I^2^ = 48%), pyelonephritis (RR 0.95, 95% CI 0.88 to 1.04) and confirmed bacterial meningitis (RR 1.02, 95% CI 0.93 to 1.11, I^2^ = 0%). The quality of the evidence was low to moderate. In conclusion, there is currently a limited body of evidence to clearly assess the clinical benefits of shorter versus longer duration antibiotics in secondary care. High quality trials assessing strategies to shorten antibiotic treatment duration for bacterial infections in secondary care settings should now be a priority.

## Introduction

The UK government [1] and WHO [2] recognise that antimicrobial resistance is one of the most important global public health threats that leads to mounting healthcare costs, treatment failure, increased morbidity and excess deaths [2,3]. Antibiotic treatment rapidly selects for resistant bacterial strains in faecal [4] and nasal flora [5,6,7]. Resistance may persist for up to a year [8] and is associated with risk of antibiotic-resistant infections [9,10], as is prior antibiotic use [10,11]. At the population level lowering antibiotic consumption is associated with lower rates of antibiotic resistance [12,13] and countries with higher antibiotic consumption tend to have higher rates of antibiotic-resistant infections [14].

In primary care, reducing antibiotic consumption can be achieved safely by avoiding or delaying prescriptions [15,16,17]. However, in secondary care patients with suspected bacterial infection are likely to be more unwell, at greater risk of poor outcomes, and more likely to benefit from antibiotic treatment [18,19]. If a patient has a life-threatening bacterial infection, delays in administering effective antibiotics of even one hour increase mortality risk [20,21]. Efforts to avoid unnecessary deaths from life-threatening bacterial infection such as Red-Flag Sepsis [22] and the new NICE sepsis guidelines [23] lower the bar for starting broad-spectrum antibiotics in the first hours or days of a patient’s illness while diagnostic information becomes available. Consequently strategies to reduce antibiotic overuse in secondary care focus on decreasing the duration and breadth of spectrum of antibiotics after they have been started. The UK Department of Health (DH) sets this approach out in its guidance ‘Start Smart then Focus’ (SSTF) [24] which recommends all antibiotic prescriptions be reviewed and revised after 24 to 72 hours with the aim of stopping or focusing treatment. However, this approach may not successfully reduce unnecessary antibiotic use [25]. Among patients who turn out not to need antibiotics, treatment may be continued as a result of ‘prescribing etiquette’, that is, clinicians’ reluctance to modify prescribing decisions previously made by others [26]. Among patients who do turn out to have a bacterial infection requiring antibiotics, recommended durations of treatment are based almost entirely on historical precedent set at a time when the dominant concern was under treatment rather than antibiotic overuse [27,28]. Clinical experience and research has progressively reduced recommended treatment [29,30] and an increasing number of primary research studies and systematic reviews of studies suggest short duration treatment may be sufficient to treat most bacterial infections. However, clinicians are hampered because this evidence is fragmented and largely contained within condition-specific reviews (e.g. meta-analyses in ventilator-associated pneumonia [31] and urosepsis [32]).

Overviews of systematic reviews involve the identification, retrieval, assessment and syntheses of the evidence from multiple systematic reviews [33]. Such reviews provide a concise synopsis of the evidence for research questions that have been addressed by systematic reviews, provide clinicians, researchers and policy makers with a succinct summary of up-to-date evidence from systematic reviews focussing on interventions for specific medical conditions, and are useful for identifying areas that future research should focus on [1,34,35].

The objective of this systematic overview was therefore to critically appraise and summarize the evidence from systematic reviews across a range of conditions comparing the effectiveness and safety of short versus long duration antibiotic treatment for the clinical resolution of bacterial infections commonly encountered in secondary care settings.

## Methods

We conducted electronic searches in the following databases: MEDLINE; Embase; Cochrane database of review of effects (DARE); Cochrane database of systematic reviews; and Cinahl. Each database was searched from inception until May 2016. No age or language restrictions were imposed. The full search strategy and list of terms is in S1 Appendix. We hand searched the bibliography of included studies to identify any other potential reviews. Where applicable, we contacted the corresponding authors of included studies for additional information [see the review protocol at http://www.crd.york.ac.uk/PROSPERO/display_record.asp?ID=CRD42016046907].

To be included in this overview, systematic reviews had to examine the clinical effectiveness of short versus long duration treatment of bacterial infections in children or adults in secondary care settings. We excluded systematic reviews of infections routinely treated or restricted to primary care and reviews of biomarker guided antibiotic therapy (because by design the intervention arms do not have a fixed duration). We also excluded reviews of tuberculosis or gastroenteritis. Studies comparing different classes of antibiotics, combination antibiotics, topical antibiotics, delayed prescriptions, or high-dose short duration versus low- or normal-dose long duration were also excluded.

Our primary outcome was clinical resolution as defined by the authors in the empirical primary studies in included reviews (clinical success, clinical resolution, clinical failure, and treatment failure). Secondary outcomes were microbiological cure, duration of symptoms, complications, adverse events, development of new mono- or multi-drug resistant species, development of antibiotic resistance, mortality, intensive therapy unit (ITU) admission and patient adherence to therapy (including after treatment). Short durations of antibiotic treatment were defined as single dose, one to three days, three to five days or five to seven days. Long duration was defined as greater than seven days, but was reported according to the duration studied for comparison to shorter durations.

Two reviewers (IJO and JC) independently screened all titles and abstracts to determine eligibility. Disagreements were resolved through discussion. Where both reviewers could not reach an agreement, a third reviewer (CCB) arbitrated. Where two or more systematic reviews were identified evaluating the same infection and with similar participants (e.g. two reviews on UTI for children), we selected one review based on the following criteria (i) most direct relevance to aim of this review, (ii) most recent and (iii) higher quality.

Where included reviews contained studies not comparing short versus long duration treatment, we included only the trials comparing short versus long duration treatment for analysis.

Quality assessment was performed using the Assessment of Multiple Systematic Reviews (AMSTAR) criteria [36] which examines 11 reporting domains of published reviews. The quality of the body of evidence across empirical studies in each included review (and for each outcome) was presented as reported by that review. We reported the overall quality of included primary studies in each review as rated by the review authors. Where an included review did not report the quality of evidence across the included primary studies or for each relevant outcome, we assessed the quality of the evidence using the Grading of Recommendation, Assessment, Development and Evaluation (GRADE) criteria [37] which examines the domains of study design, risk of bias, inconsistency, indirectness and imprecision. Three reviewers (IJO, EAS and OAG) independently assessed the quality of included reviews and the overall quality across included primary studies. Disagreements were resolved through discussion. Where reviewers were unable to reach an agreement, a third reviewer (CCB) arbitrated.

### Data extraction

Data were independently extracted by three reviewers (IJO, EAS, PST) onto customized extraction sheets according to the characteristics of the included systematic reviews. These included details about the study population, setting, diagnostic criteria, antibiotics used, short and long dosing schedules, and primary and secondary outcome measures. For each infection of interest, we used information from the source systematic review to extract data for each outcome, the study population, number of trials and participants, relative effect sizes, and quality of evidence.

We used risk ratios (RR) comparing short vs long durations with their corresponding 95% confidence intervals (95% CI) to assess the effect of interventions for dichotomous data, and mean differences (MD) with their corresponding 95% CI for continuous data. If an included review reported dichotomous outcomes using odds ratios (OR), we re-meta-analysed the data using Review Manager (RevMan) Software version 5.3 [38] to convert the OR to RR and their corresponding 95% CIs. When an included review included trials that did not meet the criteria for a specific outcome, we re-meta-analysed the data by statistically pooling data only from the relevant studies. I-square (I^2^) statistic was used to assess heterogeneity; values of 25%, 50% and 75% represented mild, moderate and substantial heterogeneity respectively. One reviewer (IJO) entered the data into RevMan, and a second reviewer (EAS) independently checked the data entry. Outcomes in adults and children were presented separately.

## Results

**Fig 1:**
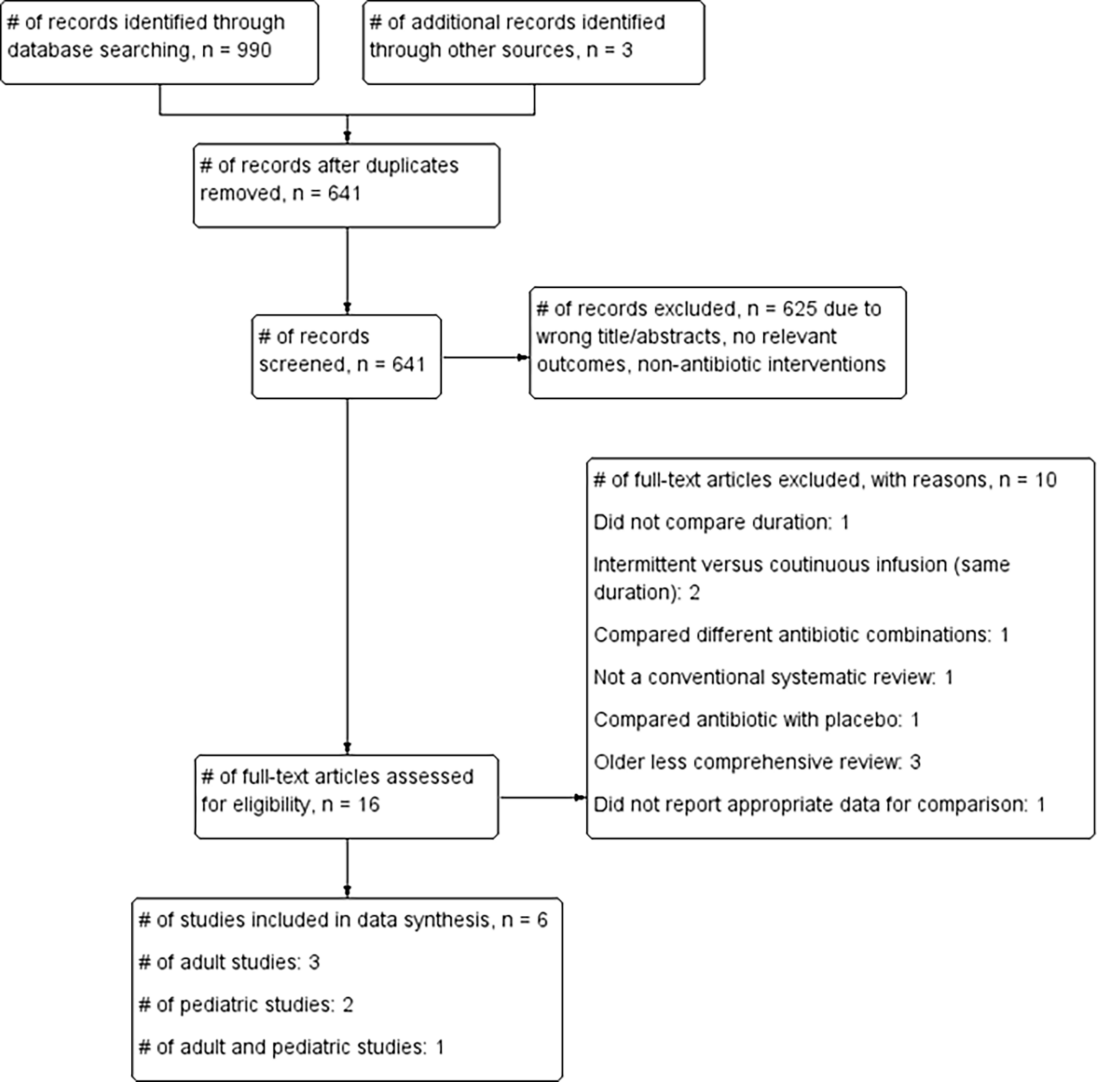
Flow chart showing the process for inclusion of systematic reviews assessing shorter versus longer duration antibiotics for bacterial infections in secondary care.

Our searches identified 641 non-duplicate citations, of which 16 articles were eligible (Fig 1). Ten articles were excluded because the report did not provide appropriate data for comparison (n = 1) [39]; the comparison was intermittent versus continuous infusion (n = 2) [40,41]; different antibiotic combinations were compared (n = 1) [42]; not a conventional systematic review (n = 1) [43]; antibiotics were compared with placebo or other interventions (n = 1) [44]; reviews were older, less comprehensive reviews of other articles assessing the same condition that was included in the overview (n = 3) [45,46,47] and because the overall duration of antibiotic therapy was similar across the intervention groups (n = 1) [48].

Six reviews [31,32,49,50,51,52] with a total of 3,162 participants were suitable for inclusion. Three reviews included only adults, two included only children, and one included both adults and children (Table 1). One included review [52] investigating short versus long duration treatment for severe community-acquired pneumonia in children did not identify any primary study for inclusion.

**Table 1:** Characteristics of included systematic reviews.

Study ID	Population	Studies (Patients)	Countries (World Bank Criteria^a^)	Diagnostic criteria	Antibiotic regimen	Definition of clinical resolution^b^	Overall quality of primary studies	AMSTAR rating^c^
Havey 2011^d^ [50]	Hospitalized patients with bacteremia or foci most commonly associated with bacteraemia (including peritonitis, pyelonephritis, pneumonia (non-ventilator associated)	Bacteremia: 1 (69); Peritonitis: 3 (240); Pyelonephritis: 1(80); Pneumonia: 6 (588)	Not specified	Laboratory	Same regimen; different duration	Not specified	Low to high^i^	Moderate
Karageorgopoulos 2009 [51]	Bacterial meningitis in children	5 (426)	USA, Greece, Switzerland, Chile, India	Clinical and laboratory	Same regimen; different duration	Complete recovery or substantial improvement of symptoms and signs of meningitis, of the per protocol patients, at the end-of-therapy evaluation	Low^j^	Moderate
Lassi 2015 [52]	Severe community-acquired pneumonia in children 2–59 months	Nil	Nil	Clinical	No restriction on the type of antibiotic; different duration	Improvement in symptoms, such as return of respiratory rate to the normal age-specific range and disappearance of chest indrawing	N/A	High
Pugh 2015 [31]	Hospital-acquired pneumonia in critically ill adults (ventilator-associated)	6 (1088)	Several^g^	Clinical and radiological	Different duration	Resolution of clinical features and improvement or lack of progression of radiographic response to therapy; or lessening of symptoms and signs of infection such that additional therapy is not required	Low to moderate^k^	High
Eliakim-Raz 2013 [32]	Acute pyelonephritis & septic UTI^e^	2 (270)	Spain, Netherlands	Clinical and laboratory	Different duration	Resolution of fever or signs and symptoms of UTI, or antibiotic modification at the end of the long-treatment arm	Low^i^	High
Chapman 2014 [49]	Pregnant women with intra-amniotic infection^f^	2 (401)	USA	Clinical	Same IV antibiotic; different duration^h^	Treatment failure defined as body temperature reading after first postpartum dose of antibiotics, either once above 39.0°C or twice above 38.4°C, at ≥4 hours apart (Edward 2003)	Low^k^	High

^a^World Bank historical classification by income (https://datahelpdesk.worldbank.org/knowledgebase/articles/906519-world-bank-country-and-lending-groups)

^b^Clinical resolution as originally defined in the review: this includes clinical success, clinical failure, or failure of treatment

^c^See web appendix table 1 for individual domain ratings

^d^6/24 were pyelonephritis (2 were exclusively hospitalized adults); 1/24 was bacteremia in hospitalized neonates; 3/24 peritonitis (all in-hospital); 13/24 were pneumonia (8 in exclusively hospitalized patients)

^e^3/8 studies exclusively in hospitals (one was different antibiotics)

^f^Only 2/11 studies short versus long-course (Edwards 1993 & Chapman 1997)

^g^One trial was an international multi-centre study conducted in 19 countries: Central and Southern America, Eastern Europe and Asia, and Western Europe, North America and Australia

^h^Antibiotics administered to all subjects diagnosed with chorioamnionitis until delivery; participants in long duration group received antibiotics for at least 48 hours, or until afebrile and asymptomatic for 24 hours

^i^Based on Cochrane risk of bias criteria. The quality rating for bacteremia was high, Peritonitis—low, Pyelonephritis–low, Pneumonia–low to moderate

^j^Based on Jadad criteria. Three studies had a score of 3, and two studies scored 2

^k^Based on GRADE rating [37]

Primary studies in the included reviews were conducted in lower-middle- to high-income settings (Table 1); however, one review [50] did not specify the setting where included primary studies were conducted. The medical conditions examined in systematic reviews involving adults included peritonitis, pyelonephritis, pneumonia and intra-amniotic infection. The conditions assessed in systematic reviews involving children were bacteraemia, pneumonia and bacterial meningitis. Five reviews defined their primary outcomes (Table 1), but only two reported definitions of their secondary outcomes (S2 Appendix). Based on the AMSTAR criteria, four reviews were rated high quality, and the remaining two were of moderate quality (Table 1) [see S1 Table for scores of individual AMSTAR domains].

### Shorter versus longer duration antibiotic treatment in adults

#### Peritonitis

**Fig 2:**
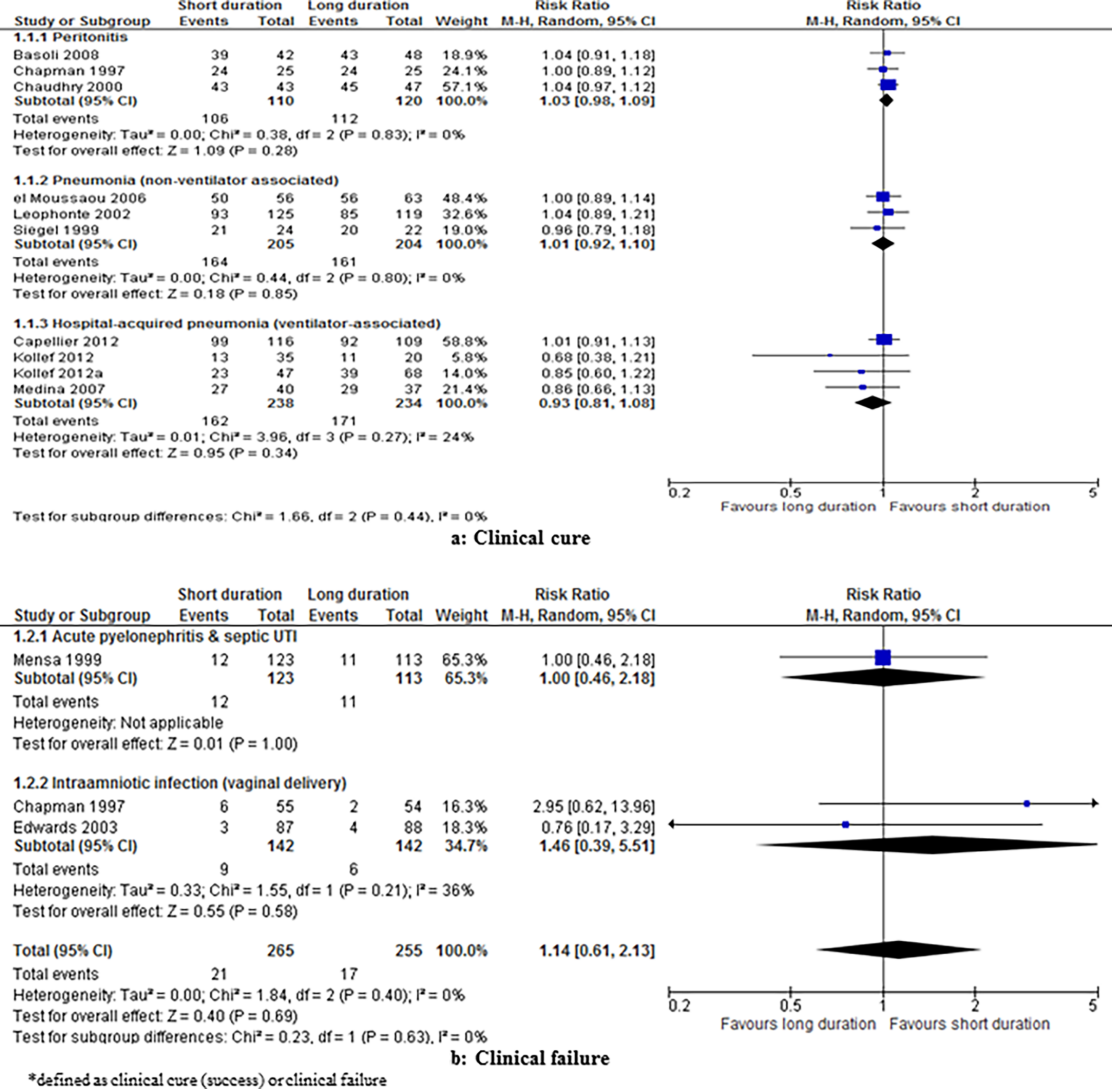
Effect of shorter versus longer duration antibiotics on clinical resolution* in adults with bacterial infection in secondary care.

We included one systematic review [50] that comprised three studies (n = 230); two of the studies included patients with spontaneous bacterial peritonitis, while the third study included patients with secondary peritonitis. The study settings were not reported. Short and long duration therapies were three to five days and ten to 14 days respectively. There was no significant difference in the rates of clinical cure between short versus long duration interventions: RR 1.03, 95% CI 0.98 to 1.09, I^2^ = 0% (Table 2; Fig 2A). The overall quality of the evidence was graded as low. There were no significant differences between short versus long duration therapy in the secondary outcomes of microbiological cure and survival (moderate quality of evidence) (Table 3).

**Table 2:** Comparison of effects of short and long duration of antibiotics in adults.

Condition	Source systematic review	Primary Outcome	Definition of short vs long duration	Studies (#)	Patients (#)	Relative effect of short compared to long duration (risk measure interpretation)	Quality of the evidence	Antibiotics used
Peritonitis	Havey 2011 [50]	Clinical cure	3–5 vs 10–14 days	3	230	RR 1.03, 95% CI 0.98 to 1.09, I^2^ = 0%, P = 0.33 (no significant difference)	Low^1^	Ertapenem; cefotaxime; cefoperazone
Pneumonia (non-ventilator-associated)	Havey 2011 [50]	Clinical cure	3–7 vs 8–10 days	3	409	^Δ^RR 1.01, 95% CI 0.92 to 1.10, I^2^ = 0%, P = 0.85 (no significant difference)	Moderate^2^	Ceftriaxone; cefuroxime; amoxicillin
Hospital-acquired pneumonia (ventilator-associated)	Pugh 2015 [31]	Clinical resolution	8+5 vs 15+5; 7 vs 10; 8 vs 12; 7 vs 16; 8 vs 15	4	472	^Δ^RR 0.93, 95% CI 0.81 to 1.08, I^2^ = 24%, P = 0.34 (no significant difference)	Moderate^3^	Beta-lactam plus aminoglycoside; carbapenems†; various‡
Acute pyelonephritis & septic UTI	Eliakim-Raz 2013 [32]	Clinical failure at EOF	7 vs 14 days	1	236	RR 1.00, 95% CI 0.46 to 2.18, P = 1.00 (no significant difference)	Moderate^4^	Ceftriaxone+cefixime; fleroxacin
		Clinical failure at EOT	7 vs 28 days	1	34	RR 1.24, 95% CI 0.49 to 3.15, P = 0.64 (no significant difference)	Very low^5^	Ampicillin or pivampicillin
Intra-amniotic infection	Chapman 2014 [49]	Failure of treatment (vaginal delivery)	Single vs multiple courses of IV antibiotics	2	284	RR 1.46, 95% CI 0.39 to 5.51; I^2^ = 36% (no significant difference)	Moderate^3^	Single vs multiple dose cefotetan (ampicillin and gentamicin during labour given to all women)* IV ampicillin and gentamycin pre-labour vs same regimen continued post-delivery**
		Failure of treatment (vaginal delivery & caesarean section)	Single vs multiple courses of IV antibiotics	1	292	RR 1.31, 95% CI 0.42 to 4.02, P = 0.64 (no significant difference)	Low^3^	Single vs multiple dose cefotetan (ampicillin and gentamicin during labour given to all women)*
		Success of treatment (caesarean section)		1	117	RR 3.31, 95% CI 0.38 to 28.75, P = 0.28 (no significant difference)	Low^3^	Single vs multiple dose cefotetan (ampicillin and gentamicin during labour given to all women)*

**Abbreviations**: EOF: end of follow-up; EOT: end of the long treatment arm; ROB: Risk of bias

*single dose of cefotetan 2 g IV within 1 hour after delivery (short duration) vs cefotetan 2 g IV every 12 hours for a minimum of 48 hours (initial dose within 1 hour after delivery) (long duration)

** IV ampicillin, 2 g every 6 hours, and gentamicin, 1.5 mg/kg every 8 hours (short duration) vs same regimen post-delivery until afebrile and asymptomatic for 24 hours (long duration)

†Doripenem 1g as 4-hour infusion 8 hourly for 7 days versus imipenem-cilastatin 1g as 1-hour infusion 8 hourly for 10 days

‡Cefoperazone- sulbactam, carbapenem and other third-generation cephalosporins; antibiotic combinations were used in 51% of cases

^Δ^Recalculated based on data from overall meta-analysis

^1^Study design: Not serious; ROB: Very serious; Inconsistency: Not serious; Imprecision: Not serious; Indirectness: Not serious

^2^Study design: Not serious; ROB: Serious; Inconsistency: Not serious; Imprecision: Not serious; Indirectness: Not serious

^3^Quality of evidence as reported from source systematic review using GRADE [37]

^4^Study design: Not serious; ROB: Serious; Inconsistency: N/A; Imprecision: Serious; Indirectness: Not serious

^5^Study design: Not serious; ROB: Very serious (open-label); Inconsistency: N/A; Imprecision: Serious; Indirectness: Not serious

**Table 3:** Secondary outcomes of systematic reviews in adults.

Condition	Source systematic review	Secondary Outcome	Definition of short vs long duration	Studies (#)	Patients (#)	Relative effect of short compared to long duration	Risk measure interpretation	Quality of the evidence
Peritonitis	Havey 2011 [50]	Microbiological cure	3–5 vs 5–14	2	154	RR 1.02, 95% CI 0.94 to 1.11, I^2^ = 0%, P = 0.66	No significant difference	Moderate^1^
		Survival	5 vs 10 days	2	140	RR 1.03 95% CI 0.97 to 1.10, I^2^ = 0%, P = 0.35	No significant difference	Moderate^2^
Pneumonia (non-ventilator-associated)	Havey 2011 [50]	Microbiological cure	3 vs 8 days	1	45	RR 1.16 95% CI 0.89 to 1.51, P = 0.28	No significant difference	Moderate^3^
Pneumonia (ventilator-associated)	Pugh 2015 [31]	28-day mortality	8 vs 15 days, 7 vs 10 days, and 7 vs 16 days	3	598	^†^RR 1.11 95% CI 0.80 to 1.55, I^2^ = 0%, P = 0.53	No significant difference	Moderate^Δ^
		Recurrence of pneumonia	8+5 vs 15+5 days; 7 vs 10 days; 8 vs 12 days; 7 vs 16 days; 8 vs 15 days	4	733	^†^RR 1.29, 95% CI 0.96 to 1.73, I^2^ = 5%, P = 0.09	No significant difference	Low^Δ^
		28-day antibiotic-free days	8 vs 15 days, 7 vs 16 days	2	431	MD 4.02 days, 95% CI 2.26 to 5.78, I^2^ = 68%, P <0.00001	Favors short course	Low^Δ^
		ITU mortality	7 vs 16 days, 8 vs 12 days	2	107	^†^RR 0.89, 95% CI 0.53 to 1.15, I^2^ = 0%, P = 0.67	No significant difference	Low^4^
		In-hospital mortality	8 vs 15 days	1	401	^†^RR 1.05, 95% CI 0.79 to 1.40, I^2^ = 0%, P = 0.74	No significant difference	Low^5^
		21-day mortality	8+5 vs 15+5 days	1	225	^†^RR1.04, 95% CI, 0.44 to 2.47, P = 0.92	No significant difference	Low^6^
		60-day mortality	8 vs 15 days	1	401	^†^RR 0.90, 95% CI 0.65 to 1.23, I^2^ = 0%, P = 0.50	No significant difference	Low^7^
		90-day mortality	8+5 vs 15+5 days	1	198	^†^RR 0.99, 95% CI 0.56 to 1.74, P = 0.97	No significant difference	Low^8^
		Relapse of pneumonia	8 vs 15 days, 8+5 vs 15+5 days	2	626	^†^RR 1.55, 95% CI 0.97 to 2.46, I^2^ = 0%, P = 0.06	No significant difference	Low^9^
		Subsequent infection due to resistant organism	8 vs 15 days	1	110	^†^RR 0.68, 95% CI 0.47 to 0.98, P = 0.04	Favors short course	Moderate^10^
		Duration of ICU stay	8 vs 15 days, 7 vs 16 days, 8+5 vs 15+5 days	3	656	MD 0.15 days, 95% CI -1.00 to 1.29, I^2^ = 0%, P = 0.80	No significant difference	Low^11^
		Duration of hospital stay	7 vs 16 days	1	30	MD -1.00 days, 95% CI -4.11 to 2.11, P = 0.53	No significant difference	Low^12^
		Duration of mechanical ventilation	7 vs 16 days, 8 vs 12 days, 8+5 vs 15+5 days	1	30	MD 0.02 days, 95% CI -0.51 to 0.54, I^2^ = 0%, P = 0.95	No significant difference	Low^13^
		Discontinuation according to CPIS; 30-day mortality	3 days vs standard course^†^	1	81	^†^RR 0.41, 95% CI 0.16 to 1.05, P = 0.06	No significant difference	Low^14^
Acute pyelonephritis & septic UTI	Eliakim-Raz 2013 [32]	Microbiological failure	7 vs 14 days	2	270	EOF: RR 0.92, 95% CI 0.61 to 1.40, I^2^ = 0%, P = 0.70	No significant difference	Low^15^
Intra-amniotic infection	Chapman 2014 [49]	Infection-related complications	Single vs multiple courses of IV antibiotics*	1	292	RR 1.87, 95% CI 0.17 to 20.37, P = 0.61 (wound infection)	No significant difference	Low^Δ^
			Single vs multiple courses of IV antibiotics*	1	292	RR 2.80, 95% CI 0.12 to 68.24, P = 0.53 (pelvic abscess)	No significant difference	Low^Δ^
		Duration of hospital stay (days)	Single vs multiple courses of IV antibiotics*	1	292	-0.9 days, 95% CI -1.64 to -0.16, P = 0.02	Favors short course	Moderate^Δ^

**Abbreviations:** CPIS: Clinical Pulmonary Infection Score; EOF: End of follow-up; ITU: Intensive therapy unit; ICU: Intensive care unit; ROB: Risk of bias

*IV ampicillin, 2 g every 6 hours, and gentamicin, 1.5 mg/kg every 8 hours vs same regimen post-delivery until afebrile and asymptomatic for 24 hours

^Δ^Quality rating as specified in the source systematic review (GRADE) [37]

†Further therapy after 3 days depended on the CPIS. The study was terminated because it was deemed “unethical”

^1^Study design: Not serious; ROB: Serious; Inconsistency: Not serious; Imprecision: Not serious; Indirectness: Not serious

^2^Study design: Not serious; ROB: Serious; Inconsistency: Not serious; Imprecision: Not serious; Indirectness: Not serious

^3^Study design: Not serious; ROB: Not serious; Inconsistency: N/A; Imprecision: Serious; Indirectness: Not serious

^4^Study design: Not serious; ROB: Serious; Inconsistency: Not serious; Imprecision: Serious; Indirectness: Not serious

^5^Study design: Not serious; ROB: Serious; Inconsistency: N/A; Imprecision: Serious; Indirectness: Not serious

^6^Study design: Not serious; ROB: Very serious; Inconsistency: N/A; Imprecision: Serious; Indirectness: Not serious

^7^Study design: Not serious; ROB: Serious; Inconsistency: Not serious; Imprecision: Serious; Indirectness: Not serious

^8^Study design: Not serious; ROB: Very serious; Inconsistency: N/A; Imprecision: Serious; Indirectness: Not serious

^9^Study design: Not serious; ROB: Serious; Inconsistency: Not serious; Imprecision: Serious; Indirectness: Not serious

^10^Study design: Not serious; ROB: Serious; Inconsistency: N/A; Imprecision: Not serious; Indirectness: Not serious

^11^Study design: Not serious; ROB: Very serious; Inconsistency: Not serious; Imprecision: Serious; Indirectness: Not serious

^12^Study design: Not serious; ROB: Very serious; Inconsistency: N/A; Imprecision: Serious; Indirectness: Not serious

^13^Study design: Not serious; ROB: Very serious; Inconsistency: Not serious; Imprecision: Serious; Indirectness: Not serious

^14^Study design: Not serious; ROB: Very serious; Inconsistency: N/A; Imprecision: Serious; Indirectness: Not serious

^15^Study design: Not serious; ROB: Very serious; Inconsistency: Not serious; Imprecision: Serious; Indirectness: Not serious

#### Pneumonia (non-ventilator-associated)

We found one systematic review [50] that comprised three studies (n = 409). The study settings were not reported. Short and long duration therapies were three to seven days and eight to 10 days respectively. There was no significant difference in the rate of clinical cure between short versus long duration therapy: RR 1.01, 95% CI 0.92 to 1.10, I^2^ = 0% (Table 2; Fig 2A). The overall quality of the evidence was moderate. There was no significant difference in microbiological cure in the one study that reported this outcome: RR 1.16, 95% CI 0.89 to 1.51. The quality of evidence of this study was moderate (Table 3).

#### Hospital-acquired pneumonia (ventilator-associated)

We found one systematic review [31] that included four studies (n = 472). The duration of antibiotic durations varied across the studies depending on the antibiotic class investigated. The study settings ranged from lower-middle- to high-income countries. There was no significant difference in the rates of clinical resolution between short versus long duration antibiotic therapy: RR 0.93; 95% CI 0.81 to 1.08, I^2^ = 24% (Table 2; Fig 2A). The overall quality of the evidence was moderate. No significant differences were reported for any secondary outcomes except for 28-day antibiotic-free days which favoured short durations: MD 4.02 days higher (2.26 to 5.78 higher); I^2^ = 68%; P <0.00001, low quality of evidence; and the risk of subsequent infection due to resistant organism which also favoured short duration treatment: RR 0.68, 95% CI 0.47 to 0.98, P = 0.04 (moderate quality of evidence) (Table 3).

#### Acute pyelonephritis and septic UTI

We included one systematic review [32] that comprised two RCTs; these compared intervention durations of seven versus 14 days and seven versus 28 days, respectively. Both studies were conducted in high-income settings. There was no significant difference in the risk of clinical failure between short versus long duration antibiotic therapy at the end of follow up in one RCT (n = 236): RR 1.00, 95% CI 0.46 to 2.18 (Table 2; Fig 2B). The quality of the evidence was moderate. The second study (n = 34) reported no significant difference in the rates of clinical failure at the end of treatment between groups: RR 1.24, 95% CI 0.49 to 3.15; however, the quality of the evidence was very low. There was no significant difference in the secondary outcome of microbiological failure: RR 0.92, 95% CI 0.61 to 1.40, I^2^ = 0% (Table 3); the quality of the evidence was low.

#### Intra-amniotic infection (pregnancy-specific condition)

We identified one systematic review [49] that included two RCTs (n = 284) which met our inclusion criteria (Table 2). Both studies were conducted in high-income settings. The studies compared single-dose versus multiple-dose antibiotics, where multiple-dose was for a minimum of 48 hours or until participants were afebrile and asymptomatic for 24 hours. There was no significant difference in the risk of treatment failure with vaginal delivery: RR 1.46, 95% CI 0.39 to 5.51; I^2^ = 36% (Fig 2B). The quality of evidence was moderate. There was no significant difference in the risk of treatment failure with caesarean section in one RCT (n = 117) that reported this outcome: RR 3.31, 95% CI 0.38 to 28.75; however, the quality of the evidence was low. There were no significant differences in the secondary outcome of infection-related complications; the quality of evidence was low (Table 3). However, the duration of hospitalisation was significantly shorter with short duration treatment in the study that reported the outcome: MD -0.9 days, 95% CI -1.64 to -0.16; the quality of evidence was moderate.

### Shorter versus longer duration antibiotic therapy in children

#### Bacteraemia

**Fig 3:**
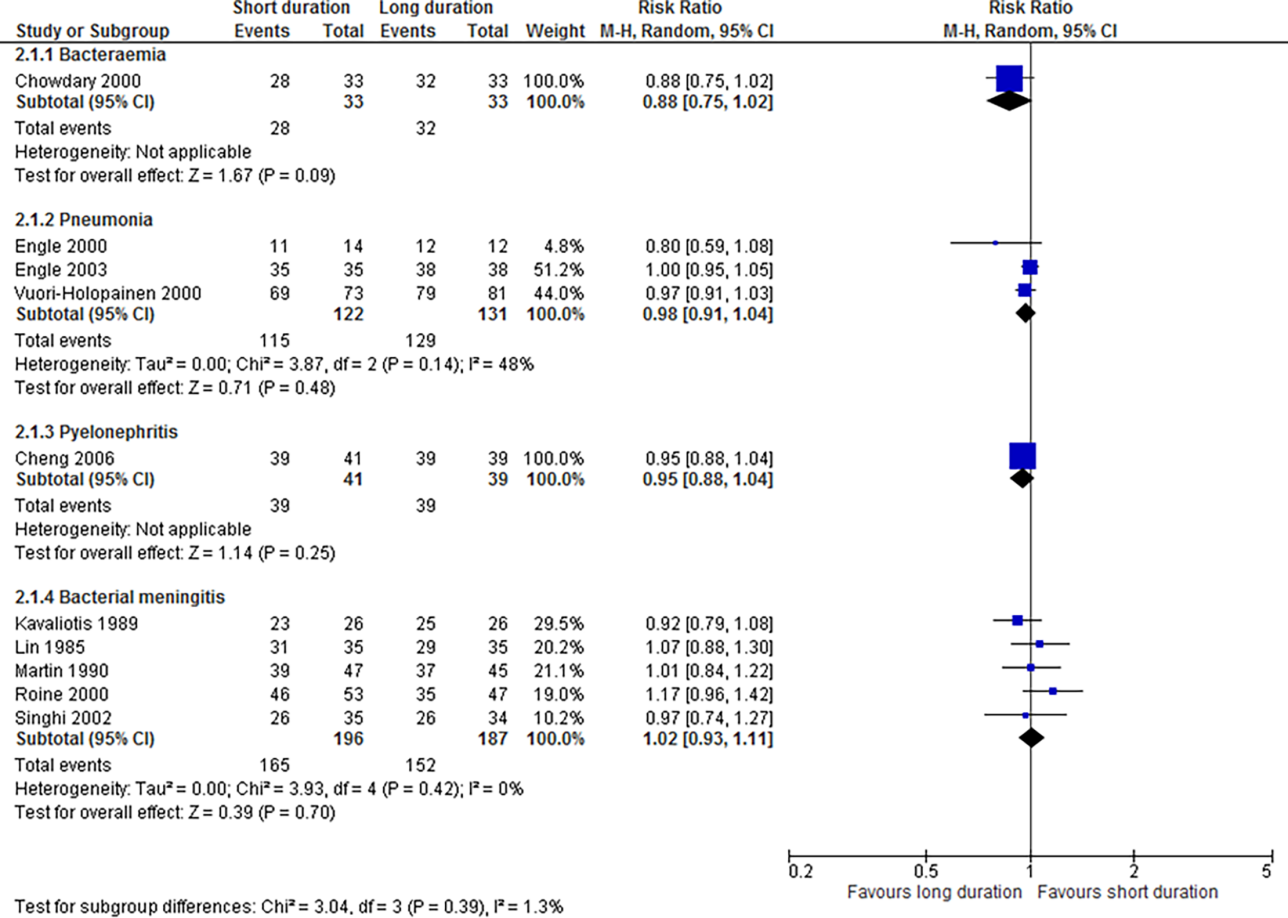
Effect of shorter versus longer duration antibiotics on clinical resolution in children with bacterial infections in secondary care.

We found one systematic review [50] that included one RCT (n = 66) which compared seven- vs 14-day antibiotic treatment. The study settings were not reported. There was no significant difference in the rates of clinical cure between short versus long duration therapy: RR 0.88, 95% CI 0.75 to 1.02 (Table 4; Fig 3). The quality of evidence was moderate. No secondary outcomes were reported.

**Table 4:** Comparison of effects of short and long duration of antibiotics in children*.

Condition	Source systematic review	Primary Outcome	Definition of short vs long duration	Studies (#)	Patients (#)	Relative effect of short versus long duration (risk measure interpretation)	Quality of the evidence	Antibiotics used
Bacteremia	Havey 2011 [50]	Clinical cure	7 vs 14 days	1	66	RR 0.88, 95% CI 0.75 to 1.02, P = 0.09 (no significant difference)	Moderate^1^	Culture-directed
Pneumonia	Havey 2011 [50]	Clinical cure	2–4 vs 4–7 days	3	253	†RR 0.98, 95% CI 0.91 to 1.04, I^2^ = 48%, P = 0.48 (no significant difference)	Low^2^	Ampicillin and gentamicin (2); Penicillin or cefuroxime (1)
Pyelonephritis	Havey 2011 [50]	Clinical cure	14 vs 21 days	1	80	RR 0.95 95% CI 0.88 to 1.04, P = 0.25 (no significant difference)	Low^3^	Culture-directed
Bacterial meningitis	Karageorgopoulos 2009 [51]	Clinical success	4–7 days vs 7–14 days	5	383	‡RR 1.02 95% CI 0.93 to 1.11, I^2^ = 0%, P = 0.70 (no significant difference)	Moderate^4^	IV ceftriaxone

**Abbreviation:** ROB: Risk of bias

*No primary studies were identified in one included systematic review [52]

†Data re-calculated using data from overall analysis

‡Data converted from OR to RR

^1^Study design: Not serious; ROB: Serious; Inconsistency: N/A; Imprecision: Not serious; Indirectness: Not serious (Chowdhary 2006)

^2^Study design: Not serious; ROB: Very serious; Inconsistency: Serious; Imprecision: Not serious; Indirectness: Not serious (Engle 2003; Engle 2000; Vuori-Holopainen 2000)

^3^Study design: Not serious; ROB: Very serious; Inconsistency: N/A; Imprecision: Not serious; Indirectness: Not serious (Cheng 2006)

^4^Study design: Not serious; ROB: Serious; Inconsistency: Not serious; Imprecision: Not serious; Indirectness: Not serious

#### Pneumonia

We found one systematic review [50] that included three RCTs (n = 253). The study settings were not reported. Short antibiotic treatment duration was two to four days, while long duration was four to seven days. There was no significant difference in the rates of clinical cure between short versus long duration therapy: RR 0.98, 95% CI 0.91 to 1.04, I^2^ = 48%, (Table 4; Fig 3); however, the quality of evidence was low.

#### Pyelonephritis

We found one systematic review [50] that included one RCT (n = 80); short and long duration therapy were 14 and 21 days respectively. The study setting was not reported. There was no significant difference in the rates of clinical cure between groups: RR 0.95, 95% CI 0.88 to 1.04 (Table 4; Fig 3). The quality of evidence was low. There was a significantly reduced rate of microbiological cure with short duration therapy in one study: RR 0.83, 95% CI 0.72 to 0.96, P = 0.01 (low quality of evidence) (Table 5).

**Table 5:** Secondary outcomes of systematic reviews in children*.

Condition	Source systematic review	Secondary Outcome	Definition of short vs long duration	Studies (#)	Patients (#)	Relative effect of short versus long duration	Risk measure interpretation	Quality of the evidence
Pyelonephritis	Havey 2011 [50]	Microbiological cure	14 vs 21 days	1	80	RR 0.83, 95% CI 0.72 to 0.96, P = 0.01	Favours longer duration	Low^a^
Bacterial meningitis	Karageorgopoulos 2009 [51]	All-cause in-hospital mortality		5	385	N/A**		
		Persistence of CSF abnormalities (pleocytosis)	4–7 vs 8–14 days	1	52	†RR 5.00, 95% CI 0.63 to 39.91, P = 0.13	No significant difference	Low^b^
		Duration of hospitalisation	7 vs 10 days	2	137	†MD: -2.95 days, 95% CI-4.79 to -1.10, I^2^ = 0%, P = 0.002	Favours shorter duration	Moderate^c^
		Total adverse events	4–7 vs 10–14 days	2	122	†RR 1.16, 95% CI 0.71 to 1.87, I^2^ = 0%, P = 0.56	No significant difference	Moderate^d^
		Withdrawals due to adverse events				NR or not observed		
		Secondary nosocomial infections	7 vs 10 days	2	139	†RR 0.51, 95% CI 0.08 to 3.14, I^2^ = 75%, P = 0.47	No significant difference	Low^e^
		Hearing impairment	4–7 vs 7–14 days	4	241	†RR: 0.74, 95% CI 0.41 to 1.32, I^2^ = 0%, P = 0.31	No significant difference	Moderate^f^
		Long-term neurological complications	4–7 days vs 7–14 days	5	367	†RR 0.70, 95% CI 0.38 to 1.30, I^2^ = 0%, P = 0.26	No significant difference	Moderate^g^

**Abbreviation:** ROB: Risk of bias; NR: Not reported

*No primary studies were identified in one included systematic review [52]

**No suitable data for meta-analysis

^a^Study design: Not serious; ROB: Very serious; Inconsistency: N/A; Imprecision: Not serious; Indirectness: Not serious

^b^Study design: Not serious; ROB: Serious; Inconsistency: N/A; Imprecision: Serious; Indirectness; Not serious

^c^Study design: Not serious; ROB: Serious; Inconsistency: Not serious; Imprecision: Not serious; Indirectness: Not serious

^d^Study design: Not serious; ROB: Serious; Inconsistency: Not serious; Imprecision: Serious; Indirectness: Not serious

^e^Study design: Not serious; ROB: Serious; Inconsistency: Very serious; Imprecision: Serious; Indirectness: Not serious

^f^Study design: Not serious; ROB: Serious; Inconsistency: Not serious; Imprecision: Serious; Indirectness: Not serious

^g^Study design: Not serious; ROB: Serious; Inconsistency: Not serious; Imprecision: Serious; Indirectness: Not serious

†Recalculated using data from source review

#### Bacterial meningitis

We found one systematic review [51] that included five RCTs (n = 383). The studies were conducted in lower-middle- to high-income settings. Antibiotic therapy was four to seven days for short duration and seven to 14 days for long duration. The bacterial organisms isolated were *Neisseria meningitidis*, *Streptococcus pneumoniae*, *Haemophilus influenzae or Streptococcus agalactiae*. There was no significant difference in the rates of clinical cure between groups: RR 1.02, 95% CI 0.93 to 1.11, I^2^ = 0% (Table 4; Fig 3). The quality of evidence was moderate. There were no significant differences in the secondary outcomes of secondary nosocomial infections or persistence of CSF abnormalities (low quality evidence). There were also no significant differences in the risk of adverse events, hearing impairment or neurological complications–moderate quality of evidence (Table 5). However, the duration of hospitalisation with significantly shorter with short duration antibiotic treatment: MD -2.95 days 95% CI -4.79 to -1.10, I^2^ = 0%, P = 0.002; the quality of evidence was moderate.

### Discussion

#### Main findings

We found six systematic reviews including 26 randomised trials of 3,162 participants to include in this overview. The data show that, for adults in secondary care, there was no difference in clinical resolution rates between short and long duration antibiotic therapy for peritonitis, pneumonia, or acute pyelonephritis and septic UTI, based on 12 studies including 1381 randomised participants, of very low to moderate quality evidence. For intra-amniotic infection there was very limited data of low to moderate quality (two trials including 292 participants) showing no difference in failure of treatment by antibiotic treatment duration. For children in secondary care, there was no difference in clinical resolution rate with short versus long duration antibiotic therapy for bacteraemia, pneumonia, pyelonephritis and bacterial meningitis, based on 10 studies of low to moderate quality including 782 randomised participants.

For adults, duration of hospitalisation was significantly shorter in intra-amniotic infection treated with short duration antibiotics. For other secondary outcomes including microbiological cure rates in peritonitis, non-ventilator-associated pneumonia, acute pyelonephritis and septic UTI, there were no important differences in secondary outcomes. For children, duration of hospitalisation was significantly shortened with short duration antibiotics in bacterial meningitis. There were no important differences in other secondary outcomes except for microbiological cure rate which was significantly lower for short versus long treatment duration in children with pyelonephritis.

Overall, there was a lack of evidence on the effect of duration of antibiotic therapy on development of new mono- or multi-drug resistance, development of antibiotic resistance, or patient adherence to therapy.

The included systematic reviews had moderate to high quality rating; however, the quality of the overall body of evidence varied from low to high. The majority of published studies to date have been conducted in high-income settings.

#### Comparison with the existing literature

We identified two overviews assessing the effect of antibiotic treatment duration on clinical outcomes. In an overview of systematic reviews for infections managed in outpatient settings [53], the authors found good evidence indicating that shorter duration therapy was as effective as longer duration for most conditions. They also found inadequate evidence about the effect of antibiotic duration on antibiotic resistance. The findings in our overview are consistent with these.

In another rapid umbrella review of shorter versus longer term antibiotic therapy for management of community acquired pneumonia in adults in secondary care [54], the author concluded that there was no significant difference in mortality rates; this is consistent with the results of our overview. Although the author of the rapid review found no evidence on the impact of antibiotic duration on duration of hospital stay, low quality evidence from our overview suggests that shortening antibiotic duration does not result in any significant impact on the duration of hospital stay (see Table 3).

#### Comparison with existing guidelines

Our findings are partly supportive of current NICE guidance for the management of hospital-acquired pneumonia in adults which specifies 5- to 10-days’ antibiotic therapy [55]; however, the results are not fully consistent with current recommendations for management of moderate to severe pneumonia (seven to 10 days). Our findings are also consistent with current American guidelines for the treatment of hospital-acquired or ventilator-associated pneumonia [56]. NICE guidance specifies 7-day ciprofloxacin therapy for patients with indwelling catheters (bladder, ureteric or nephrostomy) who have pyelonephritis [57], and the evidence from existing reviews support this; however, NICE specifies 14-day co-amoxiclav as an alternative but there is no research evidence cited for this and other recommendations.

Our findings differ from current NICE guidance which recommends 10 and 14 days of treatment for confirmed cases of *H*. *influenzae* and *S*. *pneumoniae* meningitis respectively [58]. Current American guidance recommends antibiotic duration from seven to >21 days depending on the infective organism isolated [59]. Though the evidence from our included systematic review showed that short duration treatment was as effective as long duration treatment in children, the data from the included primary studies was insufficient to assess outcomes based on causative organism.

#### Strengths and limitations

We searched extensively for systematic reviews across several sources, and we accounted for the quality of the included reviews (AMSTAR) as well as the overall quality of the evidence for each reported outcome (GRADE). However, our study has some limitations. We may not have identified all relevant reviews assessing short versus long duration antibiotic therapy in secondary care, especially unpublished articles. The variations in the definitions of primary and secondary outcomes may have contributed to the high heterogeneity observed in some of the results. In some countries, e.g. the US, many facilities provide care that qualifies as primary and secondary care; in such settings patient populations rather than settings may be more appropriate to assess the effect of antibiotic durations. In addition, the outcome results for one included review [50] is limited to patients with bloodstream infections. Overall there was little data and of variable quality, with insufficient or no data on several important outcomes. Although this overview was aimed at providing targeted audience (clinicians, microbiologists, researchers and policy makers) with up-to-date evidence, some of the included reviews did not include up-to-date primary studies.

#### Implications for clinical practice

Currently, decision-making on duration of antibiotic treatment in secondary care is hampered by a lack of evidence. The existing, limited evidence we report here supports the use of short duration antibiotic treatment for adults, but more data are needed to allow an evidence-based decision process. For children in secondary care settings with bacteraemia, pneumonia, pyelonephritis or bacterial meningitis, short durations of antibiotic therapy may attain similar rates of clinical success compared with long treatment durations, but limited evidence suggests that long treatment significantly achieves greater rates of microbiological cure in children with pyelonephritis. Short duration antibiotic treatment results in significantly shorter durations of hospitalisation.

#### Implications for research and policy

We did not identify relevant systematic reviews for several other important conditions generally managed in secondary care, including gastrointestinal/abdominal infections (except for peritonitis in adults), muscular infections, and bone and soft tissue infections. New reviews comparing such interventions are required. Newer randomised trials assessing short versus long duration antibiotics for treating bacterial infections evaluated in some of the included reviews have been published (e.g. bacterial meningitis [60], UTI [61]); consequently, the evidence for the conditions examined in those reviews needs updating. Furthermore, one included review that assessed antibiotic duration of treatment for pneumonia in children five years and under failed to identify any published primary study. Therefore, randomised trials investigating such comparisons stratified by age (for pneumonia and other childhood infections) should be conducted. Trials should be adequately powered, well-reported, and should explicitly describe outcome definitions for their primary and secondary outcomes. With the exception of the included review on bacterial meningitis in children [51], we were unable to synthesize the results on adverse events because of insufficient data. This was largely due to inadequate reporting and description of adverse events in the primary studies of the included systematic reviews. Better reporting and description of harms in future trials is therefore imperative.

None of the primary studies were conducted in a low-income setting; consequently, trials investigating the effect of antibiotic durations on health outcomes in secondary care in low-income settings should be encouraged.

Current guidelines need to be updated to reflect the current evidence base and the uncertainty within it. For example, NICE guidelines for management of confirmed bacterial meningitis in children recommends antibiotic treatment for 10–14 days in children three months and older (*H*. *influenzae* or *S*. *pneumoniae*), and 14–21 days in children under three months (Group B streptococcus, *Listeria monocytogenes*, or Gram-negative bacilli) [58]; no research document is cited for this guideline. However, the evidence base indicates that clinical outcomes were equivalent when antibiotic therapy was given for four to seven days, compared to longer durations. Future research should include an updated systematic review of all relevant up-to-date studies to evaluate the outcomes of short versus long-term antibiotics in secondary care to inform clinical practice.

## Conclusions

There is currently a limited body of systematic review evidence including highly variable quality randomised trials, to clearly assess the balance of benefit to harm of short versus long duration antibiotic treatment for bacterial infections in secondary care. This limited evidence base currently weakly supports short durations of antibiotic therapy for several conditions in adults and in children, although there is also some evidence that short antibiotic treatments are less effective than long durations at achieving microbiological cure for children with pyelonephritis. The impact of antibiotic treatment duration on the development of drug resistance in secondary care requires further research. High quality randomised trials assessing strategies to shorten antibiotic treatment duration for bacterial infections in secondary care settings should now be a priority.

## Supporting information

S1 Checklist PRISMA 2009 checklist. (PDF)

S1 Appendix Search strategies. (PDF)

S2 Appendix Definition of secondary outcomes in systematic reviews of shorter versus longer duration antibiotics in secondary care. (PDF)

S1 Table AMSTAR ratings* of included systematic reviews assessing shorter versus longer duration antibiotic therapy in secondary care. (PDF)

## References

[1] Department of Health. UK five year antimicrobial resistance strategy 2013–2018. September 2013.

[2] World Health Organisation. The evolving threat of antimicrobial resistance—Options for action 2012. http://apps.who.int/iris/bitstream/10665/44812/1/9789241503181_eng.pdf [Last accessed 24th January, 2017].

[3] European Centre for Disease Prevention and Control. Antimicrobial resistance surveillance in Europe. Annual report of the European Antimicrobial Resistance Surveillance Network (EARS-Net) 2012, 2013.

[4] Fantin B, Duval X, Massias L, Alavoine L, Chau F, Retout S, et al Ciprofloxacin dosage and emergence of resistance in human commensal bacteria. J Infect Dis. 2009 8 1;200(3):390–8. doi: 10.1086/600122 19563257 10.1086/600122 PMC2933399

[5] Chung A, Perera R, Brueggemann AB, Elamin AE, Harnden A, Mayon-White R, et al Effect of antibiotic prescribing on antibiotic resistance in individual children in primary care: prospective cohort study. BMJ. 2007 9 1;335(7617):429 doi: 10.1136/bmj.39274.647465.BE 17656505 10.1136/bmj.39274.647465.BE PMC1962897

[6] Guillemot D, Carbon C, Balkau B, Geslin P, Lecoeur H, Vauzelle-Kervroëdan F, et al Low dosage and long treatment duration of beta-lactam: risk factors for carriage of penicillin-resistant Streptococcus pneumoniae. JAMA 1998; 279(5): 365–70. 9459469 10.1001/jama.279.5.365

[7] Malhotra-Kumar S, Lammens C, Coenen S, Van Herck K, Goossens H. Effect of azithromycin and clarithromycin therapy on pharyngeal carriage of macrolide-resistant streptococci in healthy volunteers: a randomised, double-blind, placebo-controlled study. Lancet 2007; 369(9560): 482–90. doi: 10.1016/S0140-6736(07)60235-9 17292768 10.1016/S0140-6736(07)60235-9

[8] Jernberg C, Lofmark S, Edlund C, Jansson JK. Long-term ecological impacts of antibiotic administration on the human intestinal microbiota ISME J. 2007 5;1(1):56–66. doi: 10.1038/ismej.2007.3 18043614 10.1038/ismej.2007.3

[9] Ruppé E, Lixandru B, Cojocaru R, Büke C, Paramythiotou E, Angebault C, et al Relative fecal abundance of extended-spectrum-beta-lactamase-producing Escherichia coli strains and their occurrence in urinary tract infections in women. Antimicrob Agents Chemother. 2013 9;57(9):4512–7. doi: 10.1128/AAC.00238-13 23836184 10.1128/AAC.00238-13 PMC3754361

[10] Kuster SP, Rudnick W, Shigayeva A, Green K, Baqi M, Gold WL. Previous antibiotic exposure and antimicrobial resistance in invasive pneumococcal disease: results from prospective surveillance. Clin Infect Dis. 2014 10;59(7):944–52. doi: 10.1093/cid/ciu497 24973312 10.1093/cid/ciu497

[11] Costelloe C, Metcalfe C, Lovering A, Mant D, Hay AD. Effect of antibiotic prescribing in primary care on antimicrobial resistance in individual patients: systematic review and meta-analysis. BMJ 2010; 340: c2096 doi: 10.1136/bmj.c2096 20483949 10.1136/bmj.c2096

[12] Rice LB. The Maxwell Finland Lecture: for the duration-rational antibiotic administration in an era of antimicrobial resistance and clostridium difficile. Clin Infect Dis. 2008 2 15;46(4):491–6. doi: 10.1086/526535 18194098 10.1086/526535

[13] Bergman M, Huikko S, Pihlajamäki M, Laippala P, Palva E, Huovinen P, et al Effect of macrolide consumption on erythromycin resistance in Streptococcus pyogenes in Finland in 1997–2001. Clin Infect Dis. 2004 5 1;38(9):1251–6. doi: 10.1086/383309 15127336 10.1086/383309

[14] Goossens H, Ferech M, Vander Stichele R, Elseviers M, Group EP. Outpatient antibiotic use in Europe and association with resistance: a cross-national database study. Lancet 2005; 365(9459): 579–87. doi: 10.1016/S0140-6736(05)17907-0 15708101 10.1016/S0140-6736(05)17907-0

[15] Little P, Stuart B, Moore M, Coenen S, Butler CC, Godycki-Cwirko M, et al Amoxicillin for acute lower-respiratory-tract infection in primary care when pneumonia is not suspected: a 12-country, randomised, placebo-controlled trial. Lancet Infect Dis. 2013 2;13(2):123–9. doi: 10.1016/S1473-3099(12)70300-6 23265995 10.1016/S1473-3099(12)70300-6

[16] Spurling GK, Del Mar CB, Dooley L, Foxlee R, Farley R. Delayed antibiotics for respiratory infections. Cochrane Database Syst Rev. 2013 4 30;(4):CD004417 doi: 10.1002/14651858.CD004417.pub4 23633320 10.1002/14651858.CD004417.pub4

[17] Levy MM, Dellinger RP, Townsend SR, Linde-Zwirble WT, Marshall JC, Bion J, et al The Surviving Sepsis Campaign: results of an international guideline-based performance improvement program targeting severe sepsis. Crit Care Med. 2010 2;38(2):367–74. doi: 10.1097/CCM.0b013e3181cb0cdc 20035219 10.1097/CCM.0b013e3181cb0cdc

[18] Anthonisen NR, Manfreda J, Warren CP, Hershfield ES, Harding GK, Nelson NA. Antibiotic therapy in exacerbations of chronic obstructive pulmonary disease. Ann Intern Med. 1987 2;106(2):196–204. 3492164 10.7326/0003-4819-106-2-196

[19] Hersh AL, Jackson MA, Hicks LA; American Academy of Pediatrics Committee on Infectious Diseases. Principles of judicious antibiotic prescribing for upper respiratory tract infections in pediatrics. Pediatrics. 2013 12;132(6):1146–54. doi: 10.1542/peds.2013-3260 24249823 10.1542/peds.2013-3260

[20] Ferrer R, Martin-Loeches I, Phillips G, Osborn TM, Townsend S, Dellinger RP, et al Empiric antibiotic treatment reduces mortality in severe sepsis and septic shock from the first hour: results from a guideline-based performance improvement program. Crit Care Med. 2014 8;42(8):1749–55. doi: 10.1097/CCM.0000000000000330 24717459 10.1097/CCM.0000000000000330

[21] Weiss SL, Fitzgerald JC, Balamuth F, Alpern ER, Lavelle J, Chilutti M, et al Delayed antimicrobial therapy increases mortality and organ dysfunction duration in pediatric sepsis. Crit Care Med. 2014 11;42(11):2409–17. doi: 10.1097/CCM.0000000000000509 25148597 10.1097/CCM.0000000000000509 PMC4213742

[22] The UK Sepsis Trust. Appendix 1: Introducing Red Flag Sepsis. http://sepsistrust.org/wp-content/uploads/2015/08/1409314199UKSTAppendix1RFS2014.pdf [Last accessed 15 February, 2017].

[23] National Institute for Health and Care Excellence. Sepsis: recognition, diagnosis and early management NICE guidelines [NG51]. 7 2016. 32011837

[24] Public Health England. Start Smart–Then Focus. Updated March 2015. https://www.gov.uk/government/uploads/system/uploads/attachment_data/file/417032/Start_Smart_Then_Focus_FINAL.PDF.

[25] Llewelyn MJ, Hand K, Hopkins S, Walker AS. Antibiotic policies in acute English NHS trusts: implementation of 'Start Smart-Then Focus' and relationship with Clostridium difficile infection rates. J Antimicrob Chemother. 2015 4;70(4):1230–5. doi: 10.1093/jac/dku515 25538165 10.1093/jac/dku515

[26] Charani E, Castro-Sanchez E, Sevdalis N, Kyratsis Y, Drumright L, Shah N, et al Understanding the determinants of antimicrobial prescribing within hospitals: the role of "prescribing etiquette". Clin Infect Dis. 2013 7;57(2):188–96. doi: 10.1093/cid/cit212 23572483 10.1093/cid/cit212 PMC3689346

[27] Fleming WL, Holcombe MW. The effectiveness in experimental syphilis of penicillin in peanut oil-beeswax given in 16 daily injections. Am J Syph Gonorrhea Vener Dis. 1948 1;32(1):53–6. 18917625

[28] Spellberg B. The New Antibiotic Mantra-"Shorter Is Better". JAMA Intern Med. 2016 9 1;176(9):1254–5. doi: 10.1001/jamainternmed.2016.3646 27455385 10.1001/jamainternmed.2016.3646 PMC5233409

[29] Leekha S, Terrell CL, Edson RS. General principles of antimicrobial therapy. Mayo Clin Proc. 2011 2; 86(2): 156–167. doi: 10.4065/mcp.2010.0639 21282489 10.4065/mcp.2010.0639 PMC3031442

[30] File TM Jr. Duration and cessation of antimicrobial treatment. J Hosp Med. 2012;7 Suppl 1:S22–33. 23677632 10.1002/jhm.988

[31] Pugh R, Grant C, Cooke RP, Dempsey G. Short-course versus prolonged-course antibiotic therapy for hospital-acquired pneumonia in critically ill adults. Cochrane Database Syst Rev. 2015 8 24;(8):CD007577 doi: 10.1002/14651858.CD007577.pub3 26301604 10.1002/14651858.CD007577.pub3 PMC7025798

[32] Eliakim-Raz N, Yahav D, Paul M, Leibovici L. Duration of antibiotic treatment for acute pyelonephritis and septic urinary tract infection—7 days or less versus longer treatment: systematic review and meta-analysis of randomized controlled trials. J Antimicrob Chemother. 2013; 68(10): 2183–91. doi: 10.1093/jac/dkt177 23696620 10.1093/jac/dkt177

[33] McKenzie JE, Brennan SE. Overviews of systematic reviews: great promise, greater challenge. Syst Rev. 2017 9 8;6(1):185 doi: 10.1186/s13643-017-0582-8 28886726 10.1186/s13643-017-0582-8 PMC5590122

[34] Poole P, Black P. Evidence based medicine reviews. Respir Med. 2004;98:273–274.

[35] Lavis JN. How can we support the use of systematic reviews in policymaking? PLoS Med. 2009;6:e1000141 doi: 10.1371/journal.pmed.1000141 19936223 10.1371/journal.pmed.1000141 PMC2777391

[36] Shea BJ, Hamel C, Wells GA, Bouter LM, Kristjansson E, Grimshaw J, et al AMSTAR is a reliable and valid measurement tool to assess the methodological quality of systematic reviews. J Clin Epidemiol. 2009 10;62(10):1013–20. doi: 10.1016/j.jclinepi.2008.10.009 19230606 10.1016/j.jclinepi.2008.10.009

[37] Guyatt GH, Oxman AD, Vist GE, Kunz R, Falck-Ytter Y, Alonso-Coello P, et al GRADE: an emerging consensus on rating quality of evidence and strength of recommendations. BMJ. 2008 4 26;336(7650):924–6. doi: 10.1136/bmj.39489.470347.AD 18436948 10.1136/bmj.39489.470347.AD PMC2335261

[38] Review Manager (RevMan) [Computer program]. Version [5.3]. Copenhagen: The Nordic Cochrane Centre, The Cochrane Collaboration, 2014.

[39] Le Saux N, Howard A, Barrowman NJ, Gaboury I, Sampson M, Moher D. Shorter courses of parenteral antibiotic therapy do not appear to influence response rates for children with acute hematogenous osteomyelitis: a systematic review. BMC Infect Dis. 2002 8 14;2:16 doi: 10.1186/1471-2334-2-16 12181082 10.1186/1471-2334-2-16 PMC128824

[40] Yao L, MA H-X, Fan F-F, Hu L, Gao Z. Extended or continuous versus short-term intravenous infusion of meropenem/imipenem for severe infection: A meta-analysis. [Chinese]. CJEBM 2016; 16(1): 73–78.

[41] Korbila IP, Tansarli GS, Karageorgopoulos DE, Vardakas KZ, Falagas ME. Extended or continuous versus short-term intravenous infusion of cephalosporins: a meta-analysis. Expert Rev Anti Infect Ther. 2013 6;11(6):585–95. doi: 10.1586/eri.13.44 23750730 10.1586/eri.13.44

[42] Wong PF, Gilliam AD, Kumar S, Shenfine J, O'Dair GN, Leaper DJ. Antibiotic regimens for secondary peritonitis of gastrointestinal origin in adults. Cochrane Database Syst Rev. 2005 4 18;(2):CD004539 doi: 10.1002/14651858.CD004539.pub2 15846719 10.1002/14651858.CD004539.pub2 PMC11297476

[43] Dugan HA, MacLaren R, Jung R. Duration of antimicrobial therapy for nosocomial pneumonia: possible strategies for minimizing antimicrobial use in intensive care units. J Clin Pharm Ther. 2003 4;28(2):123–9. 12713609 10.1046/j.1365-2710.2003.00471.x

[44] Brodt AM, Stovold E, Zhang L. Inhaled antibiotics for stable non-cystic fibrosis bronchiectasis: a systematic review. Eur Respir J. 2014 8;44(2):382–93. doi: 10.1183/09031936.00018414 24925920 10.1183/09031936.00018414

[45] Dimopoulos G, Matthaiou DK, Karageorgopoulos DE, Grammatikos AP, Athanassa Z, Falagas ME. Short- versus long-course antibacterial therapy for community-acquired pneumonia: a meta-analysis. Drugs. 2008;68(13):1841–54. 18729535 10.2165/00003495-200868130-00004

[46] Kyriakidou KG, Rafailidis P, Matthaiou DK, Athanasiou S, Falagas ME. Short- versus long-course antibiotic therapy for acute pyelonephritis in adolescents and adults: a meta-analysis of randomized controlled trials. Clin Ther. 2008 10;30(10):1859–68. doi: 10.1016/j.clinthera.2008.10.007 19014841 10.1016/j.clinthera.2008.10.007

[47] Dimopoulos G, Poulakou G, Pneumatikos IA, Armaganidis A, Kollef MH, Matthaiou DK. Short- vs long-duration antibiotic regimens for ventilator-associated pneumonia: A systematic review and meta-analysis. Chest. 2013 12;144(6):1759–67. doi: 10.1378/chest.13-0076 23788274 10.1378/chest.13-0076

[48] Strohmeier Y, Hodson EM, Willis NS, Webster AC, Craig JC. Antibiotics for acute pyelonephritis in children. Cochrane Database Syst Rev. 2014 7 28;(7):CD003772 doi: 10.1002/14651858.CD003772.pub4 25066627 10.1002/14651858.CD003772.pub4 PMC10580126

[49] Chapman E, Reveiz L, Illanes E, Bonfill Cosp X. Antibiotic regimens for management of intra-amniotic infection. Cochrane Database Syst Rev. 2014 12 19;(12):CD010976 doi: 10.1002/14651858.CD010976.pub2 25526426 10.1002/14651858.CD010976.pub2 PMC10562955

[50] Havey TC, Fowler RA, Daneman N. Duration of antibiotic therapy for bacteremia: a systematic review and meta-analysis. Crit Care. 2011;15(6):R267 doi: 10.1186/cc10545 22085732 10.1186/cc10545 PMC3388653

[51] Karageorgopoulos DE, Valkimadi PE, Kapaskelis A, Rafailidis PI, Falagas ME. Short versus long duration of antibiotic therapy for bacterial meningitis: a meta-analysis of randomised controlled trials in children. Arch Dis Child. 2009 8;94(8):607–14. doi: 10.1136/adc.2008.151563 19628879 10.1136/adc.2008.151563

[52] Lassi ZS, Imdad A, Bhutta ZA. Short-course versus long-course intravenous therapy with the same antibiotic for severe community-acquired pneumonia in children aged two months to 59 months. Cochrane Database Syst Rev. 2015 6 16;(6):CD008032 doi: 10.1002/14651858.CD008032.pub2 26077639 10.1002/14651858.CD008032.pub2

[53] Dawson-Hahn EE, Mickan S, Onakpoya I, Roberts N, Kronman M, Butler CC, et al Short-course versus long-course oral antibiotic treatment for infections treated in outpatient settings: a review of systematic reviews. Fam Pract. 2017 9 1;34(5):511–519. doi: 10.1093/fampra/cmx037 28486675 10.1093/fampra/cmx037 PMC6390420

[54] Ghazipura M. Shorter versus longer duration of antibiotic therapy in patients with community-acquired pneumonia: a rapid review Toronto, ON: Health Quality Ontario; 2013 11 20 p. Available from: http://www.hqontario.ca/evidence/publications-and-ohtac-recommendations/rapid-reviews. [Accessed 2nd March, 2018].

[55] National Institute for Health and Care Excellence (NICE). Pneumonia in adults: diagnosis and management Clinical guideline [CG191] Published date: December 2014. https://www.nice.org.uk/guidance/cg191/chapter/1-Recommendations#hospital-acquired-pneumonia [Last accessed 1st January, 2017].

[56] Kalil AC, Metersky ML, Klompas M, Muscedere J, Sweeney DA, Palmer LB, et al Management of Adults With Hospital-acquired and Ventilator-associated Pneumonia: 2016 Clinical Practice Guidelines by the Infectious Diseases Society of America and the American Thoracic Society. Clin Infect Dis. 2016 9 1;63(5):e61–e111. doi: 10.1093/cid/ciw353 27418577 10.1093/cid/ciw353 PMC4981759

[57] National Institute for Health and Care Excellence (NICE). Clinical Knowledge Summaries. Pyelonephritis–acute. https://cks.nice.org.uk/pyelonephritis-acute#!scenariorecommendation:1 [Last accessed 1st January, 2017].

[58] National Institute for Health and Care Excellence (NICE). Meningitis (bacterial) and meningococcal septicaemia in under 16s: recognition, diagnosis and management Clinical guideline [CG102]. 1.4 Management in secondary care. Published date: June 2010 Last updated: February 2015. https://www.nice.org.uk/guidance/CG102/chapter/1-Guidance#management-in-secondary-care [Accessed 14th February, 2017].

[59] Smith L. Management of Bacterial Meningitis: New Guidelines from the IDSA. Am Fam Physician. 2005 5 15;71(10):2003–2008.

[60] Mathur NB, Kharod P, Kumar S. Evaluation of duration of antibiotic therapy in neonatal bacterial meningitis: a randomized controlled trial. J Trop Pediatr. 2015 4;61(2):119–25. doi: 10.1093/tropej/fmv002 25681965 10.1093/tropej/fmv002

[61] Darouiche RO, Al Mohajer M, Siddiq DM, Minard CG Short versus long course of antibiotics for catheter-associated urinary tract infections in patients with spinal cord injury: a randomized controlled noninferiority trial. Arch Phys Med Rehabil. 2014 2;95(2):290–6. doi: 10.1016/j.apmr.2013.09.003 24035770 10.1016/j.apmr.2013.09.003

