# Supplementary material for: Overview of systematic reviews assessing the evidence for shorter versus longer duration antibiotic treatment for bacterial infections in secondary care
Source: PLoS One. 2018 Mar 28;13(3):e0194858. doi: 10.1371/journal.pone.0194858 (PMC5874047; doi:10.1371/journal.pone.0194858)
Supplement: S1 Appendix — (PDF) [file pone.0194858.s002.pdf]

S1 Appendix: Search strategies

MEDLINE

# ▲	Searches
1	anti-infective agents/ or anti-bacterial agents/
2	(antibiotic* or anti-biotic* or antibacterial* or anti-bacterial* or antimicrobrial* or anti-microbrial* or antiinfective* or anti-infective*).ti,ab.
3	1 or 2
4	((short* or long* or standard* or prolong*) adj2 term*).ti,ab.
5	((short* or long* or standard* or prolong*) adj2 course*).ti,ab.
6	((short* or long* or standard* or prolong*) adj5 duration).ti,ab.
7	((treatment or therap*) adj3 (duration or length or day*)).ti,ab.
8	Time Factors/
9	4 or 5 or 6 or 7 or 8
10	3 and 9
11	((antibiotic* or anti-biotic* or antibacterial* or anti-bacterial* or antimicrobrial* or anti-microbrial* or antiinfective* or anti-infective*) adj5 (long* or short* or standard* or prolong*)).ti,ab.
12	((antibiotic* or anti-biotic* or antibacterial* or anti-bacterial* or antimicrobrial* or anti-microbrial* or antiinfective* or anti-infective*) adj5 (duration or length or day*)).ti,ab.
13	((antibiotic* or anti-biotic* or antibacterial* or anti-bacterial* or antimicrobrial* or anti-microbrial* or antiinfective* or anti-infective*) and (long* or short* or standard* or prolong*)).ti.
14	((antibiotic* or anti-biotic* or antibacterial* or anti-bacterial* or antimicrobrial* or anti-microbrial* or antiinfective* or anti-infective*) and (duration or length or day*)).ti.
15	10 or 11 or 12 or 13 or 14
16	Inpatients/
17	exp Hospitals/
18	Hospitalization/
19	Trauma Centers/
20	exp Intensive Care Units/
21	inpatient?.ti,ab.
22	hospital?.ti,ab.
23	hospitali?ed.ti,ab.
24	((intensive care or intensive therap* or intensive treat* or critical care or trauma) adj2 (unit? or department? or dept? or ward?)).ti,ab.
25	((intensive care or intensive therap* or intensive treat* or critical care or trauma) adj2 patient?).ti,ab.
26	16 or 17 or 18 or 19 or 20 or 21 or 22 or 23 or 24 or 25
27	15 and 26
28	Cochrane database of systematic reviews.jn. or search.tw. or meta-analysis.pt. or Medline.tw. or systematic review.tw.
29	27 and 28

EMBASE

# ▲	Searches
1	exp *antibiotic agent/ or *antiinfective agent/
2	(antibiotic* or anti-biotic* or antibacterial* or anti-bacterial* or antimicrobrial* or anti-microbrial*

	or antiinfective* or anti-infective*).ti,ab.
3	1 or 2
4	((short* or long* or standard* or prolong*) adj2 term*).ti,ab.
5	((short* or long* or standard* or prolong*) adj2 course*).ti,ab.
6	((short* or long* or standard* or prolong*) adj5 duration).ti,ab.
7	((treatment or therap*) adj3 (duration or length or day*)).ti,ab.
8	*treatment duration/
9	4 or 5 or 6 or 7 or 8
10	3 and 9
11	((antibiotic* or anti-biotic* or antibacterial* or anti-bacterial* or antimicrobrial* or anti-microbrial* or antiinfective* or anti-infective*) adj5 (long* or short* or standard* or prolong*)).ti,ab.
12	((antibiotic* or anti-biotic* or antibacterial* or anti-bacterial* or antimicrobrial* or anti-microbrial* or antiinfective* or anti-infective*) adj5 (duration or length or day*)).ti,ab.
13	((antibiotic* or anti-biotic* or antibacterial* or anti-bacterial* or antimicrobrial* or anti-microbrial* or antiinfective* or anti-infective*) and (long* or short* or standard* or prolong*)).ti.
14	((antibiotic* or anti-biotic* or antibacterial* or anti-bacterial* or antimicrobrial* or anti-microbrial* or antiinfective* or anti-infective*) and (duration or length or day*)).ti.
15	10 or 11 or 12 or 13 or 14
16	exp hospital patient/
17	exp hospital/
18	exp ward/
19	hospitalization/
20	inpatient?.ti,ab.
21	hospital?.ti,ab.
22	hospitali?ed.ti,ab.
23	((intensive care or intensive therap* or intensive treat* or critical care or trauma) adj2 (unit? or department? or dept? or ward?)).ti,ab.
24	((intensive care or intensive therap* or intensive treat* or critical care or trauma) adj2 patient?).ti,ab.
25	16 or 17 or 18 or 19 or 20 or 21 or 22 or 23 or 24
26	15 and 25
27	(meta-analysis or systematic review or MEDLINE).tw.
28	26 and 27

COCHRANE LIBRARY

ID	Search
#1	MeSH descriptor: [Anti-Bacterial Agents] explode all trees
#2	MeSH descriptor: [Anti-Infective Agents] this term only
#3	antibiotic* or anti-biotic* or antibacterial* or anti-bacterial* or antimicrobrial* or anti-microbrial* or antiinfective* or anti-infective*:ti,ab,kw (Word variations have been searched)
#4	#1 or #2 or #3
#5	((short* or long* or standard* or prolong*) near/2 term*):ti,ab,kw (Word variations have been searched)
#6	((short* or long* or standard* or prolong*) near/2 course*):ti,ab,kw (Word variations have been searched)

#7	((short* or long* or standard* or prolong*) near/5 duration):ti,ab,kw (Word variations have been searched)
#8	((treatment or therap*) near/3 (duration or length or day*)):ti,ab,kw (Word variations have been searched)
#9	MeSH descriptor: [Time Factors] explode all trees
#10	#5 or #6 or #7 or #8 or #9
#11	#4 and #10
#12	((antibiotic* or anti-biotic* or antibacterial* or anti-bacterial* or antimicrobrial* or anti-microbrial* or antiinfective* or anti-infective*) near/5 (long* or short* or standard* or prolong*)):ti,ab,kw (Word variations have been searched)
#13	((antibiotic* or anti-biotic* or antibacterial* or anti-bacterial* or antimicrobrial* or anti-microbrial* or antiinfective* or anti-infective*) near/5 (duration or length or day*)):ti,ab,kw (Word variations have been searched)
#14	((antibiotic* or anti-biotic* or antibacterial* or anti-bacterial* or antimicrobrial* or anti-microbrial* or antiinfective* or anti-infective*) and (long* or short* or standard* or prolong*)):ti (Word variations have been searched)
#15	((antibiotic* or anti-biotic* or antibacterial* or anti-bacterial* or antimicrobrial* or anti-microbrial* or antiinfective* or anti-infective*) and (duration or length or day*)):ti (Word variations have been searched)
#16	#11 or #12 or #13 or #14 or #15
#17	MeSH descriptor: [Inpatients] explode all trees
#18	MeSH descriptor: [Hospitals] explode all trees
#19	MeSH descriptor: [Intensive Care Units] explode all trees
#20	MeSH descriptor: [Hospitalization] this term only
#21	inpatient*:ti,ab,kw (Word variations have been searched)
#22	hospital or hospitals:ti,ab,kw (Word variations have been searched)
#23	hospitalized or hospitalised:ti,ab,kw (Word variations have been searched)
#24	((intensive care or intensive therap* or intensive treat* or critical care or trauma) near/2 (unit or units or department* or dept or depts or ward*)):ti,ab,kw (Word variations have been searched)
#25	((intensive care or intensive therap* or intensive treat* or critical care or trauma) near/2 (patient or patients)):ti,ab,kw (Word variations have been searched)
#26	#17 or #18 or #19 or #20 or #21 or #22 or #23 or #24 or #25
#27	#16 and #26

CINAHL

#	Query
1	(MH "Antibiotics+")
2	TI (antibiotic* or anti-biotic* or antibacterial* or anti-bacterial* or antimicrobrial* or anti-microbrial* or antiinfective* or anti-infective*) OR AB (antibiotic* or anti-biotic* or antibacterial* or anti-bacterial* or antimicrobrial* or anti-microbrial* or antiinfective* or anti-infective*)
3	S1 OR S2
4	TI (((short* or long* or standard* or prolong*) N2 term*)) OR AB (((short* or long* or standard* or prolong*) N2 term*))
5	TI (((short* or long* or standard* or prolong*) N2 course*)) OR AB (((short* or long* or standard* or prolong*) N2 course*))

6	TI (((short* or long* or standard* or prolong*) N5 duration*)) OR AB (((short* or long* or standard* or prolong*) N5 duration*))
7	TI (((treatment or therap*) N3 (duration or length or day*))) OR AB (((treatment or therap*) N3 (duration or length or day*)))
8	(MH "Treatment Duration")
9	S4 OR S5 OR S6 OR S7 OR S8
10	S3 AND S9
11	TI (((antibiotic* or anti-biotic* or antibacterial* or anti-bacterial* or antimicrobrial* or anti-microbrial* or antiinfective* or anti-infective*) AND (long* or short* or standard* or prolong*))) OR AB (((antibiotic* or anti-biotic* or antibacterial* or anti-bacterial* or antimicrobrial* or anti-microbrial* or antiinfective* or anti-infective*) N5 (long* or short* or standard* or prolong*)))
12	TI (((antibiotic* or anti-biotic* or antibacterial* or anti-bacterial* or antimicrobrial* or anti-microbrial* or antiinfective* or anti-infective*) AND (duration or length or day*))) OR AB (((antibiotic* or anti-biotic* or antibacterial* or anti-bacterial* or antimicrobrial* or anti-microbrial* or antiinfective* or anti-infective*) N5 (duration or length or day*)))
13	S10 OR S11 OR S12
14	(MH "Inpatients")
15	(MH "Hospitals+")
16	(MH "Hospitalization")
17	(MH "Intensive Care Units+")
18	TI inpatient* OR AB inpatient*
19	TI (hospital OR hospitals) OR AB (hospital OR hospitals)
20	TI (hospitalized OR hospitalised) OR AB (hospitalized OR hospitalised)
21	TI (((intensive care or intensive therap* or intensive treat* or critical care or trauma) N2 (unit or units or department* or dept or depts or ward or wards))) OR AB (((intensive care or intensive therap* or intensive treat* or critical care or trauma) N2 (unit or units or department* or dept or depts or ward or wards)))
22	TI (((intensive care or intensive therap* or intensive treat* or critical care or trauma) N2 (patient OR patients))) OR AB (((intensive care or intensive therap* or intensive treat* or critical care or trauma) N2 (patient OR patients)))
23	S14 OR S15 OR S16 OR S17 OR S18 OR S19 OR S20 OR S21 OR S22
24	S13 AND S23
25	PT meta-analysis OR MH meta-analysis OR TI (meta-analysis OR metaanalysis OR systematic review OR medline) OR AB (meta-analysis OR metaanalysis OR systematic review OR medline) OR PT review
26	S24 AND S25
