# Supplementary material for: Overview of systematic reviews assessing the evidence for shorter versus longer duration antibiotic treatment for bacterial infections in secondary care
Source: PLoS One. 2018 Mar 28;13(3):e0194858. doi: 10.1371/journal.pone.0194858 (PMC5874047; doi:10.1371/journal.pone.0194858)
Supplement: S2 Appendix — (PDF) [file pone.0194858.s003.pdf]

S2 Appendix: Definition of secondary outcomes in systematic reviews of short versus long duration antibiotics in secondary care.

A. Systematic reviews of studies including only adult subjects

Study	Condition	Secondary outcomes	Definition
Havey 2011 [50]	Peritonitis	Microbiological cure	Not reported
		Survival	Not reported
	Pneumonia (non-ventilator-assisted)	Microbiological cure	
Pugh 2015 [31]	Hospital-acquired pneumonia in critically ill adults (including VAP)	28-day mortality	- all-cause mortality
		Recurrence of pneumonia	Clinical and/or microbiological criteria
		28-day antibiotic-free days	- total number of antibiotic-free days over a 28-day period from onset of VAP and initiation of antibiotics
		ITU mortality	Not reported
		In-hospital mortality	Not reported
		21-day mortality	Not reported
		60-day mortality	Not reported
		90-day mortality	Not reported
		Relapse of pneumonia	Not reported
		Subsequent infection due to resistant organism	Not reported
Eliakim-Raz 2013 [32]	Acute pyelonephritis & septic UTI	Microbiological failure*	-bacteriological recurrence (≥10 cfu per mL) with no symptoms from the urinary tract at short-term follow-up (asymptomatic bacteriuria left untreated)<br>- persistence of original uropathogen, clinical failure<br>- superinfection with a new uropathogen, persistence of original uropathogen<br>- superinfection with a new uropathogen, persistence of original uropathogen (cure: $<10^3$)<br>- persistence of original uropathogen<br>- superinfection with a new uropathogen, persistence of original uropathogen
Chapman 2014 [49]	Intra-amniotic infection	Infection-related complications	Not reported
		Duration of hospital stay (days)	Not reported

B. Systematic reviews of studies including only pediatric subjects.

Study	Condition	Secondary outcomes	Definition
Havey 2011 [50]	Pyelonephritis	Microbiological cure	Not reported
Karageorgopoulos 2009 [51]	Bacterial meningitis	All-cause in-hospital mortality	Not reported
		Persistence of CSF abnormalities (pleocytosis)	Not reported
		Duration of hospitalisation	Not reported
		Total adverse events	Not reported
		Withdrawals due to adverse events	Not reported
		Secondary nosocomial infections	Not reported
		Hearing impairment	Not reported
		Long-term neurological complications	Not reported

*as defined in included primary studies

Abbreviations: CFU: colony-forming unit; CSF: cerebrospinal fluid; VAP: Ventilator-assisted pneumonia
