# Supplementary material for: Overview of systematic reviews assessing the evidence for shorter versus longer duration antibiotic treatment for bacterial infections in secondary care
Source: PLoS One. 2018 Mar 28;13(3):e0194858. doi: 10.1371/journal.pone.0194858 (PMC5874047; doi:10.1371/journal.pone.0194858)
Supplement: S1 Table — (PDF) [file pone.0194858.s004.pdf]

S1 Table: AMSTAR ratings* of included systematic reviews assessing short versus long duration antibiotic therapy in secondary care.

**AMSTAR Criteria**	Havey 2011 [50]	Karageorgopoulos 2009 [51]	Lassi 2015 [52]	Pugh 2015 [31]	Eliakim-Raz 2013 [32]	Chapman 2014 [49]
Was an 'a priori' design provided?	0	0	1	1	0	1
Was there duplicate study selection and data extraction?	?	0	1	1	1	1
Was a comprehensive literature search performed?	1	1	1	1	1	1
Was the status of publication (i.e. grey literature) used as an inclusion criterion?	0	1	1	1	1	1
Was a list of studies (included and excluded) provided?	0	0	1	1	0	1
Were the characteristics of the included studies provided?	1	1	1	1	1	1
Was the scientific quality of the included studies assessed and documented?	1	1	1	1	1	1
Was the scientific quality of the included studies used appropriately in formulating conclusions?	1	1	1	1	1	1
Were the methods used to combine the findings of studies appropriate?	1	1	1	1	1	1
Was the likelihood of publication bias assessed?	0	1	1	1	1	1
Was the conflict of interest stated?	1	1	1	?	1	1
**Total score**	**6**	**8**	**11**	**10**	**9**	**11**

* Score domains:1 = Yes; 0 = No; ? = Unclear

*Overall quality of systematic review: 0-4 = low quality; 5-8 = moderate quality; 9-11 = high quality
